# Are KLIC and CRIME-80 scores useful to assist decision-making initially or at the moment of repeat DAIR? – a retrospective study

**DOI:** 10.5194/jbji-10-403-2025

**Published:** 2025-10-28

**Authors:** Joana Contente, Carlos Ferreira, Mário Silva, Guilherme Madeira, Ana Ribau, Ricardo Sousa

**Affiliations:** 1 Orthopaedics 2 Department, Centro Hospitalar de Leiria EPE, Leiria, 2410-197, Portugal; 2 Orthopaedics Department, Unidade Local de Saúde de Santo António EPE, Porto, 4099-001, Portugal; 3 Orthopaedics Department, Hospital Distrital de Santarém EPE, Santarém, 2005-177, Portugal; 4 Orthopaedics Department, Centro Hospitalar do Médio Ave EPE, Vila Nova de Famalicão, 4760-107, Portugal; 5 Porto Bone and Joint Infection Group (GRIP), Unidade Local de Saúde de Santo António EPE and Hospital Lusíadas, Porto, 4099-001, Portugal

## Abstract

**Introduction**: Debridement, antibiotics, and implant retention (DAIR) is recommended for early acute postoperative and late acute periprosthetic joint infections (PJIs). The KLIC and CRIME-80 scores have been proposed to predict DAIR outcomes. Nevertheless, their clinical utility remains uncertain. This study aimed to evaluate their predictive value for DAIR failure in our cohort, both at primary indication and when repeat DAIR was considered necessary. **Methods**: We retrospectively reviewed all patients who underwent DAIR for total hip or knee PJI between 2010 and 2021, with at least 1 year of follow-up. Failure was defined as persistent infection, need for implant removal, amputation, or infection-related death. **Results**: A total of 102 patients were included, with a mean follow-up of 48.9 months. The overall failure rate was 35.3 %. Failure rates did not differ significantly between patients who underwent a single DAIR and those who required repeat procedures (32.5 % vs. 45.5 %, p= 0.26). No significant correlations were found between KLIC or CRIME-80 scores and failure rates, either at the initial indication for DAIR ( p= 0.54 and p= 0.93, respectively) or in repeat DAIR procedures ( p= 0.44 and p= 0.50, respectively). **Conclusions**: In our cohort, the KLIC and CRIME-80 scores were not predictive of DAIR failure, either at initial treatment or when repeat DAIR was required. These scores offered limited prognostic value and did not support clinical decision-making. Prospective studies are needed to validate and improve predictive tools for DAIR outcomes.

## 1 Introduction

A periprosthetic joint infection (PJI) is a severe complication of total hip and knee arthroplasties, with incidence increasing alongside rising procedure numbers (Kuiper et al., 2013). PJIs can be classified as early acute postoperative, chronic, or late acute (LA). Early infections occur mostly within 3 months after implantation, and LA cases typically result from documented or presumed hematogenous spread at a later stage (Zimmerli et al., 2004). These infections are significantly challenging to treat, leading to considerable patient morbidity and financial burden on healthcare systems (Chalmers et al., 2019; Kurtz et al., 2018; Shahi et al., 2017).

Treatment aims to eradicate infection while maintaining joint function and mobility. Options include antibiotic suppression, implant removal, one- or two-stage revision, arthrodesis, amputation, and surgical debridement, antibiotics, and implant retention (DAIR). DAIR is an attractive option as it involves a single surgery, avoids implant removal morbidity, shortens disability time, and, if successful, is potentially more cost-effective than two-stage revision (Byren et al., 2009). Current guidelines support DAIR for both early and LA PJI, particularly when the implant is stable and the pathogen is sensitive to anti-biofilm therapies (Sigmund et al., 2025).

DAIR success rates vary widely, with lower efficacy seen in LA infections. (Aboltins et al., 2013; Bergkvist et al., 2016; Duque et al., 2017; Kuiper et al., 2013; Sukeik et al., 2012; Urish et al., 2018; Vilchez et al., 2011). Several factors (including patient condition, infection severity, symptom duration, possibility to exchange modular components, and pathogen type) can influence the prognosis (Sigmund et al., 2025; Wouthuyzen-Bakker et al., 2020). DAIR failure may compromise future revision outcomes, particularly two-stage revisions (Lizaur-Utrilla et al., 2019; Tsang et al., 2017), making accurate patient selection essential to avoid ineffective interventions (Duffy et al., 2018; Löwik et al., 2018; Rajgopal et al., 2018; Wouthuyzen-Bakker et al., 2019). To aid this selection, the KLIC and CRIME-80 scoring systems were developed to predict DAIR failure in early acute and LA PJIs, respectively (Tornero et al., 2015; Wouthuyzen-Bakker et al., 2019).

To date, evidence supporting the KLIC and CRIME-80 scores as decision-making tools in clinical practice remains limited and inconsistent. In our practice, these scores are not used routinely to guide DAIR indication but are considered when evaluating repeat DAIR procedures. Our main goal was to assess their predictive value for DAIR failure in our clinical practice. We aimed to look at two different clinical decision-making moments: (a) primary indication for a DAIR and (b) when a repeat DAIR is clinically judged necessary.

## 2 Methods

### 2.1 Study design

We retrospectively reviewed all patients submitted to DAIR for PJI following hip or knee arthroplasty at our institution between 2010 and 2021, with a minimum follow-up of 1 year. PJI was defined according to the 2021 European Bone and Joint Infection Society criteria (McNally et al., 2021). LA PJI was defined as the development of acute symptoms occurring ≥ 3 months after implantation in a previously asymptomatic joint. All DAIRs were performed for presumed acute infections after primary or revision surgery. Exclusion criteria were tumour-related joint replacements and incomplete medical records.

### 2.2 Data collection

Data were collected from institutional electronic records, including (1) demographics; (2) co-morbidities (chronic kidney failure (CKF), liver cirrhosis, chronic obstructive pulmonary disease (COPD), rheumatoid arthritis (RA), diabetes, and malignancy); (3) initial surgical characteristics: site, indication (primary or secondary osteoarthritis, rheumatoid arthritis, arthroplasty in femoral head fractures, other fractures, and infection), type (primary, aseptic revision, two-stage revision for infected joint, one-stage revision for infected joint), cementation; (4) C-reactive protein (CRP) level; (5) DAIR procedure dates and polyethylene exchange; (6) microbiological results; (7) antibiotic treatment; (8) outcomes (success or failure) and type of failure; and (9) follow-up.

### 2.3 Our clinical practice

Surgical treatment was performed by different surgeons within the department and not exclusively by infection-dedicated surgeons. Arthrocentesis with joint fluid analysis (cell count, biochemistry, microbiology) was performed whenever feasible. Empirical intravenous (IV) antibiotics, usually vancomycin and piperacillin/tazobactam, were initiated postoperatively and adjusted once culture and sensitivity results were available. IV therapy was usually continued for 7–10 d before switching to oral antibiotics if the patient's clinical and laboratory response was favourable.

Repeat DAIR was performed on an “as needed” basis, when there was clinical concern that the infection remained uncontrolled following the initial procedure (e.g. persistent wound leakage, local inflammatory signs, or persistently high CRP). Decisions were made by a multidisciplinary team, considering known poor prognostic factors, such as surgery >6 weeks in early acute postoperative or duration of symptoms >7 d in LA PJI, positive blood cultures, extremely elevated CRP, anti-biofilm antibiotic-resistant microorganisms, significant patient co-morbidities, and the possibility for a liner exchange.

### 2.4 Outcome measures

Failure was defined as infection persistence (with or without suppressive antibiotics), the need for subsequent implant removal, amputation, or infection-related death. When used, suppressive antibiotics were continued in the long term, indefinitely. The need for repeat DAIR was not necessarily considered a failure, as long as the infection was ultimately considered cured without the need for subsequent implant removal or suppressive antibiotic therapy. The KLIC score was calculated for early acute PJIs, and CRIME-80 was calculated for LA PJI. Patients were then categorised into groups according to their KLIC score ( ≤ 2, 2.5–3.5, 4–5, 5.5–6.5, and ≥ 7) and to their CRIME-80 score ( - 1, 0, 1–2, 3–4, and > 5), respectively.

### 2.5 Statistical analysis

Descriptive statistics ( n , %, min, max, mean, standard deviation) were calculated for the study variables. The groups were compared using the Fisher test and the Pearson chi-squared test (qualitative variables) and with a t test or Mann–Whitney U (quantitative variables). Variables that reached statistical significance or were considered clinically relevant ( p<0 .02) were included in a logistic regression and multivariable analysis. For all inferential statistics, a probability of type I error of 0.05 was considered (95 % confidence interval).

## 3 Results

A total of 102 patients were included in this study. The mean patient age was 68 years, with 57.8 % female. Common co-morbidities were diabetes (29.4 %), CKF (9.8 %), and COPD (8.8 %) (Table 1). The mean CRP at presentation was 132 mg L^−1^.

**Table 1:** Patient characteristics ( n = 102).

	n	% of total	Min	Max	Mean (SD)
Sex					
Male	43	42.2			
Female	59	57.8			
Age (yr)			28	94	68.3 (11.1)
Co-morbidities					
CKF	10	9.8			
Liver cirrhosis	6	5.9			
COPD	9	8.8			
RA	8	7.8			
Diabetes	30	29.4			
Malignancy	7	6.9			

Overall, 52.0 % of patients were diagnosed with hip PJI, and 48.0 % were diagnosed with knee PJI. The initial surgical indication was osteoarthritis in 69.6 % of patients, femoral head fractures in 13.7 % of patients, other fractures in 4.9 % of patients, and previous PJI in 7.8 % of patients. Although 8 patients were diagnosed with rheumatoid arthritis, only 4 (3.9 %) underwent initial surgery due to this condition, as 3 underwent surgery following a fracture and 1 underwent surgery after PJI. Considering the type of initial surgery, 76.5 % of patients were initially submitted to primary arthroplasty, 14.7 % of patients were submitted to aseptic revision, 7.8 % of patients were submitted to infected two-stage revision surgery, and 1.0 % of patients were submitted to infected one-stage revision surgery. After the beginning of symptoms, the time to first DAIR was < 1 week in 54.9 % of patients, between 1 and 2 weeks in 29.4 % of patients, between 2 and 4 weeks in 10.8 % of patients, and > 6 weeks in 3.9 % of patients. Repeat DAIR was performed in 21.6 % of patients. In total, 72.5 % of patients were diagnosed as early acute PJIs, and 27.5 % of patients were diagnosed as LA PJIs.

**Table 2:** Surgical data.

	n	% of total
Infection site		
Hip	53	52.0
Knee	49	48.0
Initial surgical indication		
Primary or secondary osteoarthritis	71	69.6
Rheumatoid arthritis	4	3.9
Arthroplasty in femoral head fractures	14	13.7
Other fractures	5	4.9
Infection	8	7.8
Initial surgery		
Primary	78	76.5
Aseptic revision	15	14.7
Two-stage revision of infection	8	7.8
One-stage revision of infection	1	1.0
Initial cementation	70	68.6
Time until first DAIR		
< 1 week	56	54.9
1–2 weeks	30	29.4
2–4 weeks	11	10.8
4–6 weeks	0	0.0
> 6 weeks	4	3.9
Polyethylene exchange	101	99.0
Repeat DAIR	22	21.6
Early acute PJI	74	72.5
LA PJI	28	27.5

The mean KLIC score was 3.2 points, and the mean CRIME-80 score was 1.4 points (Table 3). No patients had missing variables to complete the scoring system.

**Table 3:** Score categorisation.

	M (SD)	Success, n	Failure, n	p
KLIC score	3.2 (1.48)			
≤ 3.5		27	10	0.451
> 3.5		24	13	
CRIME-80 score	1.4 (1.57)			
< 3		12	10	0.843
≥3		3	3	

The overall DAIR failure rate was 35.3 % ( 36/102 ). Implant removal occurred in 26.5 % of patients, infection persistence was managed conservatively in 4.9 % of patients, and infection-related death occurred in 3.9 % of patients. No amputations were reported (Table 4). Suppressive antibiotics were used in 3.9 % of patients, with a mean follow-up of 4 years.

The failure rate was not significantly different between single and repeated DAIR: 32.5 % ( 26/80 ) vs. 45.5 % ( 10/22 ) ( p=0 .26). The DAIR failure rate was also not significantly different between early acute postoperative or LA PJI: 31.1 % ( 23/74 ) vs. 46.4 % ( 13/28 ) ( p=0 .169).

**Table 4:** DAIR failure and the distribution of failure types across KLIC and CRIME-80 scores.

	KLIC		CRIME-80		Total	
	n	%	n	%	n	%
Implant removal	16	15.6	11	10.8	27	26.5
Amputation	0	0.0	0	0.0	0	0.0
Infection-related death	3	2.9	1	1.0	4	3.9
Infection persistence (with or without suppressive antibiotics)	3	2.9	2	2.0	5	4.9
Overall failure rate					36	35.3
Failure after 1 DAIR					26	32.5^a^
Failure after repeated procedure					10	45.5^b^

^a^ Percentage of the total number of patients submitted to 1 DAIR. ^b^ Percentage of the total number of patients submitted to repeated DAIR.

No significant correlations were found between KLIC or CRIME-80 scores and DAIR failure rates ( p= 0.54 and p= 0.93, respectively). Patients with successful DAIR procedures presented all ranges of previous KLIC scores, without a strong correlation in any stratified group. No predictive risk threshold was identified for KLIC or CRIME-80.

**Figure 1:**
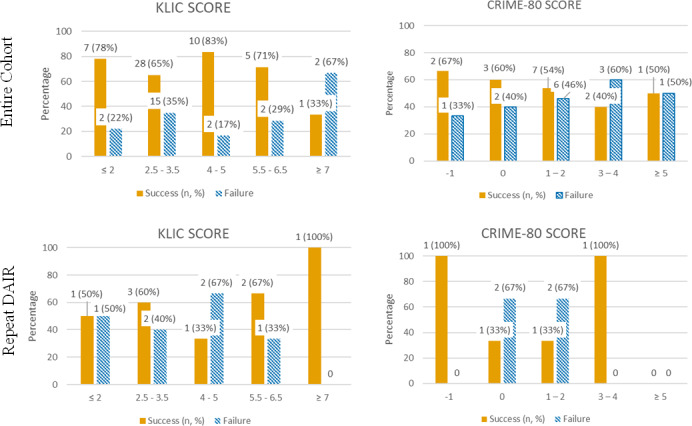
KLIC and CRIME-80 score distribution in the entire cohort and in patients that needed to repeat DAIR (orange: patients with a successful DAIR procedure; striped blue: patients with a failed DAIR procedure).

Focusing specifically on the repeated DAIR cohort, the KLIC and CRIME-80 scores were also not associated with failure ( p = 0.44 and p = 0.50, respectively) (Fig. 1).

Of all registered variables, the only independent risk factor significantly associated with failure was CRP level (OR = 1.004, 95 % CI: 1.000–1.008). DAIR failure increases by 1.04 for each 10 mg L^−1^ CRP rise. However, the effect size was modest (Nagelkerke $R^{2}$ = 0.363). Despite the statistical significance of CRP, its limited predictive value diminishes its clinical usefulness as a standalone marker. Therefore, in our study, no reliable predictive factors for DAIR failure could be identified (Table 5).

**Table 5:** Univariable and multivariable analysis of DAIR failure risk factors.

Risk factors	Success	Failure	Univariable	Multivariable
	n (%) or	n (%) or	p	p (OR CI 95 %)
	median (IQR)	median (IQR)		$R^{2}$ = 0.363
Female	37 (56.1)	22 (61.1)	0.622	–
Age	69 (14)	69 (14)	0.766	–
Body mass index	28.04 (6)	28.85 (9)	0.812	–
Diabetes	21 (31.8)	9 (25)	0.470	–
Malignancy	5 (7.6)	2 (5.6)	0.700	–
COPD	6 (9.1)	3 (8.3)	0.897	–
CKF	6 (9.1)	4 (11.1)	0.743	–
Liver cirrhosis	3 (4.5)	3 (8.3)	0.437	–
RA	2 (3)	6 (16.7)	0.014	0.089 (0.775–35.740)
Joint replaced (hip)	29 (43.9)	24 (66.7)	0.028	0.160 (0.114–1.431)
Polyethylene exchange	61 (92.4)	30 (83.3)	0.157	0.370 (0.080–2.565)
Bacteremia	11 (16.7)	5 (13.9)	0.712	–
Microorganism defined as “difficult to treat”	9 (15.8)	2 (6.3)	0.189	0.439 (0.08–2.990)
LA PJI	51 (77.3)	23 (63.9)	0.148	0.519 (0.150–2.608)
Polymicrobial samples	25 (37.9)	11 (30.6)	0.460	–
CRP	64.0 (154.5)	134.5 (238.8)	0.053	0.050 (1.000–1.008)
Antibiotic (days)	97(93)	83 (82)	0.156	–
IV antibiotic (days)	10 (12)	19.5 (18)	0.006	0.098 (0.992–1.103)
Symptomatic time to DAIR	19 (16.3)	21 (18.8)	0.983	–
Score cutoff: KLIC > 3 or CRIME-80 ≥ 3	27 (40.9)	16 (44.4)	0.730	0.696 (0.215–2.252)
Adequate anti-biofilm antibiotic	51 (77.3)	19 (52.8)	0.014	0.191 (0.138–1.484)

## 4 Discussion

In our cohort, neither the KLIC scores nor the CRIME-80 scores reliably predicted DAIR failure in early or late acute PJI, even when repeat DAIR procedures were performed. No meaningful risk thresholds were identified, including among patients with high KLIC scores ( ≥ 7), contrary to previous reports (Duffy et al., 2018). Similarly, KLIC scores > 3.5 did not achieve an optimal balance of sensitivity and specificity, conflicting with Tornero et al. (2015). CRIME-80 scores ≥ 3 also failed to predict failure, in contrast with Wouthuyzen-Bakker et al. (2019), who reported a preoperative CRIME-80 ≥ 3 as an independent predictor.

Several studies have reported clinical utility of the KLIC score (Argenson et al., 2019; Duffy et al., 2018; Jiménez-Garrido et al., 2018), although methodological heterogeneity may explain discrepancies. Inconsistent definitions of failure (Duffy et al., 2018; Jiménez-Garrido et al., 2018) and the inclusion of acute hematogenous infections (Jiménez-Garrido et al., 2018) may have influenced outcomes. Lowik et al (2018) also supported KLIC as a predictor, though with lower sensitivity, specificity, and accuracy than initially described (Tornero et al., 2015), likely due to their retrospective study design and cohort differences, underlining the importance of external validation before widespread clinical adoption.

Recent external validations have yielded conflicting results. In an Australasian cohort, both KLIC and CRIME-80 scores were validated as predictors of DAIR failure (Hoffman et al., 2024). However, in a northern European population, Liukkonen et al. (2024) found the KLIC score unreliable for predicting early DAIR failure. These discrepancies may reflect differences in co-morbidity profiles, highlighting the need to assess predictive models within a specific population.

Our findings align with studies questioning the predictive value of the KLIC score (Bernaus et al., 2022; Chalmers et al., 2021). Although Chalmers et al. (2021) reported moderate predictive value for the CRIME-80 score at 90 d and 2 years, methodological limitations were noted. Antibiotic suppression was not classified as DAIR failure, and the use of low thresholds (CRIME–80 > 1 or adjusted ≥ 0) reduced clinical utility, as nearly any patient with minimal risk factors (e.g. male sex or age > 80) was categorised as high risk. Additionally, this study attempted validation in a US population, where confounding demographic and clinical factors may have influenced outcomes.

Surgical heterogeneity may also compromise predictive accuracy. Boyer and Cazorla (2021) noted higher DAIR success in hip arthroplasties compared with knee arthroplasties. Since the KLIC score includes parameters unequally distributed between joint types (e.g. cemented prostheses, fracture indications), bias may be introduced. The authors therefore propose stratifying score models by joint type. Evidence suggests that the infecting microorganism, especially streptococcal species, and modular exchange are stronger predictors of outcome in LA PJIs (Lora-Tamayo et al., 2017; Sabater-Martos et al., 2020; Wouthuyzen-Bakker et al., 2020). These studies reinforce the importance of isolating the causative microorganism before surgery. However, they are rarely known preoperatively and are thus not integrated into existing scoring systems.

To our knowledge, this is the first study to evaluate these scores in the context of repeated DAIR procedures. Predictive performance was even poorer in this subgroup, reinforcing that their use in guiding surgical decisions should be approached with caution.

Study limitations include a retrospective design, small sample size, lack of a control group, and potential selection and observer bias from surgeries performed by different surgeons. Because of its sample size, this study may have insufficient statistical power to detect small to moderate negative findings. Despite its novelty, the repeat DAIR subgroup is also relatively small, which reduces the statistical power to detect meaningful associations. Data were obtained from a single tertiary care referral centre, potentially limiting generalisability. Three surgeons were involved in patients' follow-up and data collection, medical records may be incomplete, and pathology distribution was unequal, which may further introduce bias. Additionally, a tendency for lower risk classification in both scoring systems could affect result interpretation.

## 5 Conclusions

PJI remains a serious complication after hip and knee arthroplasty. While DAIR is commonly recommended for early and late acute cases, KLIC and CRIME-80 scores lack widespread clinical adoption and external validation. In our cohort, neither score predicted DAIR failure, indicating limited prognostic utility and small clinical value as predictive models for DAIR outcomes. Their performance was particularly unreliable in patients undergoing repeated DAIR interventions, suggesting that these scores should not guide surgical decision-making in such cases. Further prospective research with larger samples is needed to validate these scores or to develop more reliable predictive clinical tools to support clinical decisions and improve outcomes.

## Data Availability

The datasets used during the current study are available from the corresponding author on reasonable request.
